# The importance of pharmacists in modern day surgery – editorial

**DOI:** 10.1097/JS9.0000000000000146

**Published:** 2023-02-16

**Authors:** Andrew A. Wireko, Pearl Ohenewaa Tenkorang, Favour Tope Adebusoye, Owusu Yaa Asieduwaa, Aashna Mehta, Anastasia Fosuah Debrah, Victor Nkemsinachi Oti, Jyi Cheng Ng, Toufik Abdul-Rahman, Vladyslav Sikora

**Affiliations:** a Sumy State University, Sumy; b Kyiv Medical University, Bytom Campus, Poland; c Faculty of Pharmacy and Pharmaceutical Sciences, Kwame Nkrumah University of Science and Technology, Kumasi; d University of Ghana Medical School, Accra, Ghana; e Faculty of Medicine, University of Debrecen, Debrecen, Hungary; f Faculty of Medicine and Health Sciences, University of Putra, Selangor, Malaysia

Surgical care delivery, which consists primarily of surgical personnel and physical resources, is an important aspect of health care that has evolved significantly over time. The importance of a multidisciplinary healthcare (MDH) approach in modern healthcare delivery, particularly surgery, cannot be overstated due to the enormous benefits and value it has added to patient care. Since the introduction of concepts of pharmaceutical care and drug therapy in surgical services in the 1980s, the concept of integrating pharmacists in surgical services has not been widely utilized^1^,^2^. Nonetheless, pharmacists’ primary responsibilities back then were medication distribution and regulatory compliance, but this has changed in recent years^1^. Clinical pharmacists (CPs) have been well integrated into healthcare systems, particularly in many developed nations, but the need for CPs in surgical care, unlike other subspecialties, is not well discussed in the literature.

Several studies have found that drug administration errors are the most common in surgical delivery, particularly during anesthesia care^3^. According to a study conducted by Nanji *et al*.^3^, 5.3% of medication administrations during 277 surgeries were incorrect, and 79.3% were avoidable. A number of recent papers have discussed the critical importance of CPs in surgical procedures, and they are primarily trained in detecting and correcting these errors associated with drug administration^2^,^4^. CPs have the potential to collaborate with surgeons and make meaningful contributions. Other significant benefits of CP integration into surgical care include improving the quality of care, limiting patient costs, and, most importantly, reducing mortality^4^.

This editorial aims to highlight the primary roles of CPs in surgical care, as well as their benefits, challenges, and recommendations.

## The roles and benefits of CPs in surgery

Perioperative enhanced recovery programs have been established in recent years and have significantly influenced surgical care around the world^5^. One notable strategy used by these enhanced recovery programs is the inclusion of CPs and pharmacologists in surgical procedures to achieve desired results^5^. The role of CPs during surgical procedures and even in other specialties is unquestionably necessary for efficient healthcare delivery and positive patient outcomes^4^–^6^. The Perioperative Clinical Pharmacist has advanced therapeutic knowledge and experience in order to ensure appropriate medication use and patient outcomes throughout the surgical procedure. They are also responsible and accountable for correcting any medication-related issues that may arise during the preoperative, intraoperative, and postoperative phases for all surgical patients^6^. During the preoperative period, the CP ensures fluid status optimization, appropriate analgesia administration, antimicrobial prophylaxis, and venous thromboembolism prophylaxis^6^. At the intraoperative stage, the CP ensures that antimicrobial prophylaxis, fluid resuscitation, and anesthetic plans are redosed. Opioid doses are adjusted based on the patient’s tolerance and adverse effects^6^. During the postoperative period, the patient receives medication monitoring, withdrawal of strong opioids, and counseling.

The CPs offer a unique perspective on the multidisciplinary nature of perioperative teams and the impact of their collaboration on the outcome of surgical procedures^4^,^5^. Due to their training and knowledge in medication safety, CPs can comprehend and participate in the analysis of the root causes of surgical complications^5^. Furthermore, CPs are skilled at implementing a systematic approach to surgical care, which includes the acquisition, sorting, and evaluation of patient data by actively collecting medication-related data and keeping records^5^. They are also well-positioned to educate surgical patients about what to expect as a result of exposure to certain drugs that may be used prior to, during, and after surgical procedures^5^,^7^. The CP intervention during the surgical procedure, according to Luo *et al*., amounted to providing extended educational sessions and a booklet containing the pharmacokinetics, pharmacodynamics, indications, duration, and dose of stress ulcer prophylaxis to the surgical residents who prescribe acid suppressants and the nurses who dispense them. In addition, CPs collected information about surgical patients from electronic medical records and hospital information systems to analyze the appropriate use of prophylactic acid suppressants and other medications in terms of dose, duration, combination, and replacement therapy^7^.

The majority of these studies highlighting the need for CPs have discussed the enormous value and benefits that CPs bring to surgical teams. Some of the main benefits of CP integration include reduced drug administration errors, adverse drug effects, shorter hospital stays, lower costs, and many more^4^,^5^. This is supported by Leape *et al*.^8^, who found that integrating critical care pharmacists reduced adverse drug effects by 66%, saving USD 270,000 per year. Another study conducted in a Canadian hospital found that all patients admitted for surgery saved between CA$1518 and CA$3048 with CP integration^4^. A review of an Enhanced Recovery After Surgery value-based surgery revealed that the presence of a pharmacist saved approximately €153–€6537 per colorectal case^9^. A systematic review of studies in southern Asia found that CP interventions can improve therapeutic and safety outcomes for patients, as well as humanistic outcomes such as disease knowledge and treatment adherence^10^. For example, Ahmed *et al*.^10^ found that CP interventions significantly reduced the odds of medication errors by 73%. Upadhyay *et al*. also found that CP interventions significantly decreased the direct healthcare costs of patients, with a greater reduction in drug costs and investigation costs. Bond and Raehl^11^ further highlighted that pharmacist involvement in surgical antibiotic prophylaxis led to a 52.1% lower mortality rate, a 10.2% reduction in length of stay, and a 34.3% lower incidence of postoperative infections. Additionally, 90% of physicians surveyed by Moreno *et al*.^12^ agreed that pharmacists’ recommendations are clinically helpful, and pharmacists have increased their knowledge of the medications they prescribe. These findings emphasize the importance of pharmacist involvement in reducing medication errors and costs in all populations.

## Is there any regard for CPs in the clinical setting and surgical delivery in low-income and middle-income countries (LMICs)?

Several studies have demonstrated that interprofessional collaboration and a collaborative practice framework improve overall patient outcomes^13^. As a result, healthcare providers, particularly physicians and pharmacists, must work together to improve healthcare and overall patient outcomes. However, multidisciplinary collaborations for effective surgical delivery are uncommon and are only used in a few LMICs. This is primarily due to a misunderstanding of pharmacists’ and other nonphysician essential roles in healthcare delivery, particularly surgery^14^,^15^. This is reflected in the belief that pharmacists may provide incorrect prescription information, illegal refills, and/or inappropriate comments to patients. According to an Alipour poll, three-fifths (63.1%) of respondents believed that pharmacists should not be given the authority to write repeat prescriptions on their own^14^. CPs are undervalued and underrepresented in surgical delivery and multidisciplinary teams globally, particularly in LMICs^16^. A study in an Ethiopian hospital discovered a 76% drug therapy problem in surgical wards, highlighting the importance of CPs during surgical procedures^2^. The typical role of CPs is poorly understood, as evidenced by a study in which most LMICs believe that pharmacist training is typically geared toward the industrial sector^15^.

Noninvolvement of CPs in LMICs increases the risk of medication errors, inadequate pain and symptom management, and inadequate management of postsurgical complications^17^–^19^, which can have serious consequences such as adverse drug reactions, prolonged hospital stays, and even high mortality. There is a shortage of trained healthcare workers who are qualified to administer medications in surgical settings in many LMICs. Furthermore, there is a huge disparity between health workers and patients, which makes physicians prone to mistakes and errors, which can have serious consequences for patients^20^.

The major obstacles to the effective practice of incorporating CPs during surgeries differ from one geographical location to the next^15^. Many LMICs lack trained CPs who are qualified to work in surgical settings. Malaysia, for example, has a pharmacist-to-patient ratio of 1 : 6207, and Ghana has only 619 pharmacists serving a population of 2.9 million in their capital region, Accra^15^, which falls far short of the WHO recommendation of 1 : 2000.

Furthermore, CPs are frequently not fully integrated into surgical teams, and in many cases, they are not involved in decision-making nor consulted about medication management during surgery. Many LMICs lack surgical clinical pharmacy facilities, including a lack of space and equipment, as well as limited access to necessary resources, such as medications and surgical supplies, making it difficult for CPs to provide the level of care that patients require^15^,^21^.

## Recommendations

It is evident that CPs have a tremendous opportunity to improve clinical outcomes, particularly surgical care. However, a number of countries, particularly LMICs, do not meet the WHO recommendation of a pharmacist-to-patient ratio of 1 : 2000. All countries, particularly LMICs, should prioritize training more CPs to work in clinical and surgical settings to improve patient care. To encourage pharmacists to pursue advanced clinical training, nations should prioritize developing a career structure that supports clinical development and specialization in practice. Institutions should also include more clinical content and experiential learning opportunities in the undergraduate pharmacy training curriculum and conduct these at clinical pharmacy service locations. More funding for postgraduate clinical pharmacy programs would help pharmacists improve their clinical skills and confidence in clinical settings.

MDH approaches should be widely encouraged and adopted globally, particularly in LMICs, to achieve high-quality results in patient care. Pharmacists should be included in MDH approaches because most CP engagements in healthcare settings have yielded excellent results thus far.

Governments should invest in the development of clinical pharmacy facilities in surgical settings, as well as in the provision of necessary resources such as medications and surgical supplies. Nonphysician healthcare providers, such as CPs, should be fully integrated into surgical teams and involved in decision-making and medication management during surgery. Stakeholders, including doctors and other healthcare professionals, should be more aware of pharmacists’ expanded role in patient care and the value of clinical pharmacy practice.

Given the scarcity of data on the outcomes of CPs integration into surgical settings, more research should be conducted to provide additional confirmation on CPs impacts.

## Ethical approval

Not applicable.

## Sources of funding

None.

## Author contribution

A.A.W.: conceptualized ideas. All authors were involved in the process of data curation, writing of the initial draft, and review and editing. All authors approved the final manuscript.

## Conflicts of interest disclosure

There are no conflicts of interest.

## Research registration unique identifying number (UIN)

1. Name of the registry: NA.

2. Unique identifying number or registration ID: NA.

3. Hyperlink to your specific registration (must be publicly accessible and will be checked): NA.

## Guarantor

Andrew Awuah Wireko.

## Consent for publication

Not applicable.

## Data availability

No new data were generated.

## References

[1] Bickham P Golembiewski J Meyer T . ASHP guidelines on perioperative pharmacy services. Am J Health Syst Pharm, 76 2019:903–20. 31361851 10.1093/ajhp/zxz073

[2] Tefera GM Zeleke AZ Jima YM . Drug therapy problems and the role of clinical pharmacist in surgery ward: prospective observational and interventional study. Drug Healthc Patient Saf 2020;12:71–83. 32440225 10.2147/DHPS.S251200 PMC7210033

[3] Nanji KC Patel A Shaikh S . Evaluation of perioperative medication errors and adverse drug events. Anesthesiology 2016;124:25–34. 26501385 10.1097/ALN.0000000000000904 PMC4681677

[4] Neville HL Chevalier B Daley C . Clinical benefits and economic impact of post-surgical care provided by pharmacists in a Canadian hospital. Int J Pharm Pract 2013;22:216–22. 23952872 10.1111/ijpp.12058

[5] Lovely JK Hyland SJ Smith AN . Clinical pharmacist perspectives for optimizing pharmacotherapy within Enhanced Recovery After Surgery (ERAS®) programs. Int J Surg 2019;63:58–62. 30665004 10.1016/j.ijsu.2019.01.006

[6] Patel GP Hyland SJ Birrer KL . Perioperative clinical pharmacy practice: responsibilities and scope within the surgical care continuum. J Am Coll Clin Pharm 2020;3:501–19.

[7] Luo H Fan Q Xiao S . Impact of clinical pharmacist interventions on inappropriate prophylactic acid suppressant use in hepatobiliary surgical patients undergoing elective operations. PLoS One 2017;12:e0186302. 29045435 10.1371/journal.pone.0186302 PMC5646810

[8] Leape LL Cullen DJ Clapp MD . Pharmacist participation on physician rounds and adverse drug events in the intensive care unit. JAMA 1999;282:267–70. 10422996 10.1001/jama.282.3.267

[9] KRS-Knowledge Resource Service. KRS Website: Surgery: Enhanced Recovery After Surgery ERAS. Accessed 16 December 2022. https://krs.libguides.com/surgery/eras

[10] Ahmed A Saqlain M Tanveer M . The impact of clinical pharmacist services on patient health outcomes in Pakistan: a systematic review. BMC Health Serv Res 2021;21:859. 34425816 10.1186/s12913-021-06897-0 PMC8381566

[11] Bond CAC Raehl CL . Clinical and economic outcomes of pharmacist-managed antimicrobial prophylaxis in surgical patients. Am J Health Syst Pharm 2007;64:1935–42. 17823105 10.2146/ajhp060631

[12] Moreno G Lonowski S Fu J . Physician experiences with clinical pharmacists in primary care teams. J Am Pharm Assoc 2003;57:686–91. 10.1016/j.japh.2017.06.018 28811089

[13] Parrish RH Bodenstab HM Carneal D . Positive patient postoperative outcomes with pharmacotherapy: a narrative review including perioperative-specialty pharmacist interviews. J Clin Med 2022;11:5628. 36233497 10.3390/jcm11195628 PMC9572852

[14] Alipour F Peiravian F Mehralian G . Perceptions, experiences and expectations of physicians regarding the role of pharmacists in low-income and middle-income countries: the case of Tehran hospital settings. BMJ Open 2018;8:e019237. 10.1136/bmjopen-2017-019237 PMC5829860 29420231

[15] Azhar S Hassali MA Ibrahim MIM . The role of pharmacists in developing countries: the current scenario in Pakistan. Hum Resour Health 2009;7:54. 19594916 10.1186/1478-4491-7-54 PMC2714831

[16] Rojas-Gutierrez E Vilar-Compte D . An overview of surgical site infection in low- and middle-income countries: the role of recent guidelines, limitations, and possible solutions. Curr Treat Options Infect Dis 2019;11:300–16.

[17] Abbasi S Rashid S Khan FA . A retrospective analysis of peri-operative medication errors from a low-middle income country. Sci Rep 2022;12:12404. 35858974 10.1038/s41598-022-16479-7 PMC9300725

[18] Walters JL Jackson T Byrne D . Postsurgical pain in low- and middle-income countries. Br J Anaesth 2016;116:153–5. 26787783 10.1093/bja/aev449

[19] Viktil KK Blix HS . The impact of clinical pharmacists on drug-related problems and clinical outcomes. Basic Clin Pharmacol Toxicol 2008;102:275–80. 18248511 10.1111/j.1742-7843.2007.00206.x

[20] Abbasi S Rashid S Khan FA . A retrospective analysis of peri-operative medication errors from a low-middle income country. Sci Rep 2022;12:12404. 35858974 10.1038/s41598-022-16479-7 PMC9300725

[21] Morris AM Lee CN-H . Common issues in the unique environment of global surgical health services research. JAMA Surg 2018;153:979–80. 30046811 10.1001/jamasurg.2018.1981

